# Physical, Mechanical, and Microstructure Characteristics of Ultra-High-Performance Concrete Containing Lightweight Aggregates

**DOI:** 10.3390/ma16134883

**Published:** 2023-07-07

**Authors:** Aref A. Abadel

**Affiliations:** Department of Civil Engineering, College of Engineering, King Saud University, Riyadh 11421, Saudi Arabia; aabadel@ksu.edu.sa

**Keywords:** durability, lightweight aggregates, thermal analysis, rheology, SEM analysis

## Abstract

This study explores and enhances the resistance of an ultra-high-performance concrete (UHPC) to explosive spalling under elevated temperatures. This study investigates the impact of lightweight aggregates (LWAs) on the mechanical and microstructural properties of the UHPC. Various UHPC specimens were created by replacing silica sand with LWAs in percentages ranging from 0% to 30%. The evaluation of these specimens involved assessing their compressive and flexural strengths, density, mass loss, shrinkage, porosity, and microstructural characteristics using scanning electron microscopy (SEM). This study provides valuable insights by analyzing the influence of lightweight aggregates on the strength, durability, and microstructure of UHPC. The results reveal that incorporating LWAs in the UHPC improved its flowability while decreasing its density, as the percentage of LWAs increased from 5% to 30%. Including 30% LWA resulted in a mass loss of 4.8% at 300 °C, which reduced the compressive and flexural strengths across all curing durations. However, the UHPC samples subjected to higher temperatures displayed higher strength than those exposed to ambient conditions. The microstructure analysis demonstrated that the UHPC specimens with 30% LWA exhibited increased density due to continuous hydration from the water in the lightweight aggregate. The pore size distribution graph indicated that incorporating more of the LWA increased porosity, although the returns diminished beyond a certain point. Overall, these findings offer valuable insights into the influence of lightweight aggregates on the physical and strength characteristics of UHPC. This research holds significant implications for developing high-performance, lightweight concrete materials.

## 1. Introduction

Ultra-high-performance concrete has emerged as a revolutionary material in construction due to its exceptional mechanical properties, high durability, and resistance to various environmental factors [1]. Incorporating lightweight fine aggregates (LWFAs) in UHPC is a promising strategy for reducing its density and enhancing performance [2]. LWFAs are known for reducing the weight of concrete while maintaining its strength and durability, making them ideal candidates for use in UHPC [3]. UHPC, also called reactive powder concrete, is a novel cement composite with outstanding mechanical properties and durability [4,5,6]. Nevertheless, UHPC has a low water-to-cement ratio (*w*/*c*) that can lead to less than 50% cement hydration. Consequently, the UHPC matrix may have many un-hydrated binder particles that do not contribute to developing hardening properties [7]. The low w/b ratio in UHPC causes notable autogenous shrinkage, which can result in cracking [8]. Additionally, self-desiccation increases the capillary tension of pore water, leading to autogenous shrinkage [9,10]. Therefore, it is essential to provide more water to expedite the cement hydration process and reduce self-desiccation. The UHPC mixture’s high impermeability makes external water-curing techniques inefficient since it is difficult for water to penetrate the concrete matrix and contribute to cement hydration [11]. In addition to enhancing the mechanical and durability properties of UHPC, using LWCAs can also significantly affect its microstructural properties [12,13,14]. The porous nature of LWCAs allows them to act as internal curing agents, which leads to a more homogenous and denser microstructure of UHPC. This reduces the number and size of voids in the matrix, which improves the overall mechanical and durability performance of UHPC [15,16,17]. Lura et al. [18] suggest that including a few internal curing agents that can efficiently disperse in the matrix is a more active approach than reserving water when blending and setting concrete and then slowly discharging the water for internal curing. Varga et al. [19] used a pre-saturated lightweight aggregate (LWA) to achieve internal curing and decrease the shrinkage of concrete. However, past research on ultra-high-performance concrete that utilized internal curing observed a trade-off amid physical characteristics and the shrinkage of concrete when using a mineral admixture [20,21].

High-strength lightweight concrete (HSLC) is often produced using lightweight aggregates for structural applications [22]. These aggregates contain pores, which lead to a 20–40% reduction in the weight of the HSLC mixture compared to traditional concrete [23]. In a study by Khayat et al. [24], the effectiveness of internal curing with different amounts of an LWA in the UHPC matrix was examined. They discovered that substituting 25% of the LWA volume caused the greatest strength, albeit with a decreased elastic modulus in the UHPC, similar to what is observed in high-performance concrete. The effectiveness of LWCAs on the strength, durability, and microstructural properties of UHPC has been the subject of extensive research in recent years. Several studies have investigated the impact of different types of LWCAs, such as expanded clay, pumice, and perlite, on the mechanical properties of UHPC. These studies have demonstrated that using LWCAs can improve the compressive strength, tensile strength, and flexural strength of UHPC. Furthermore, the addition of LWCAs has been shown to increase the resistance of UHPC to freeze–thaw cycles, chemical attacks, and abrasion.

Prior research has assessed the performance of UHPCs that underwent internal curing with LWAs, such as prewetted calcined bauxite [25], pumice [26], expanded shale [27], and permeable fine aggregate. Some UHPCs displayed a decline in strength compared to their reference mixtures. In contrast, some research involving well-designed internal curing agents revealed marginally higher strength without considering the elastic modulus [28,29]. Weiss et al. [30] stated that a favorable internal curing effect could be achieved in concrete composites by carefully designing mixture proportions that account for the water absorbed by the LWA before the cement setting. Hu et al. [31] investigated the impact of an LWA’s physicochemical properties on strength formation in UHPC. An overview of existing studies revealed that incorporating LWAs in concrete mixes improved the properties at the macroscale. Consequently, it is anticipated that integrating LWAs into the UHPC system will affect its resistance to high temperatures. Upon examining the literature above, it is evident that there is a scarcity of research focusing on the strength and microstructural characteristics of ultra-high-performance concrete comprising LWAs at heating conditions. Wei and Liu et al. [32,33] found that when a bauxite aggregate is combined in an air-dry state, it can absorb water during the blending process and release it into the concrete mix after setting. This demonstrates the capacity of this bauxite aggregate to facilitate internal curing in UHPC mixtures. Various techniques for offering water for internal curing in the concrete matrix have been documented in prior research [34,35]. The most common methods involve using additional internal curing water for prewetting or incorporating dry agents with internal curing water during blending. Alternatively, some strategies maintain the same water quantity as the reference mix.

When exposed to high temperatures, concrete spalls are likely influenced by various factors, such as concrete materials, aggregate type, aggregate size, specimen dimensions, heating rate, loading conditions, and testing methods [36,37]. Although UHPC possesses remarkable mechanical properties and durability, it is more susceptible to explosive spalling than conventional concrete under elevated temperatures [38,39]. So et al. [40] documented violent spalling in UHPC at temperatures of 500 degrees Celsius during 10 min of heating. Richard et al. [41] observed reduced free water in UHPC at elevated temperatures of 250 degrees Celsius. This observation was attributed to the possible buildup of xonotlite and crystal hydrate, which increased the pozzolanic reaction achievement phase to 94% at a heating condition, then 72% at an ambient condition, ultimately resulting in a dense microstructure for the UHPC. Kodur [38] and Amran [39] described the violent spalling process at high temperatures. It has been proposed that explosive spalling in UHPC mixtures occurs due to the buildup of high pore pressure. The denser microstructures of UHPC result in internal water vapor pressure formation at elevated heat, increasing the likelihood of violent spalling as porosity decreases due to the low *w*/*b* ratio [40]. Furthermore, Phan et al. [42] examined the violent spalling of HPC subjected to temperatures of 235 degrees Celsius, attributing it to thermal stress and internal pore pressure.

In recent decades, researchers have investigated developing ultra-high-performance concrete mixes by adding fillers or admixtures to achieve superior mechanical and durability properties when the UHPC is subjected to various situations [43,44]. The recent inclusion of lightweight aggregates (LWAs) in the UHPC matrix has been investigated. While studies have examined the strength and durability characteristics of UHPC-containing LWAs, limited research has focused on the microstructure, thermal analysis, pore structure, and porosity of ultra-high-performance lightweight concrete.

*Originality Aspect of Paper*: This study aims to comprehensively evaluate the physical, strength, and microstructural attributes of ultra-high-performance concrete (UHPC) compositions with varying proportions of lightweight aggregates (LWAs). By introducing different dosages of LWAs into the UHPC matrix, the influence of LWA content on the engineering properties of the mixture was systematically examined. Scanning electron microscopy and X-ray diffraction analysis were conducted on the UHPC-LWA blend to delve deeper into the microstructure. Incorporating LWAs into UHPC holds great promise for enhancing the mechanical and durability properties of construction projects while simultaneously promoting sustainability. This research seeks to contribute to the existing knowledge by shedding light on the impact of LWA content on the performance of UHPC under elevated temperatures. Consequently, it aims to facilitate the development of optimized UHPC mixtures that meet the multifaceted requirements of diverse structural applications. Ultimately, this investigation endeavors to pave the way for producing highly durable, sustainable, and high-performance concrete composites, which will undoubtedly find extensive utilization within the construction industry.

## 2. Experimental Program

### 2.1. Materials

#### Cement

**Figure 1:**
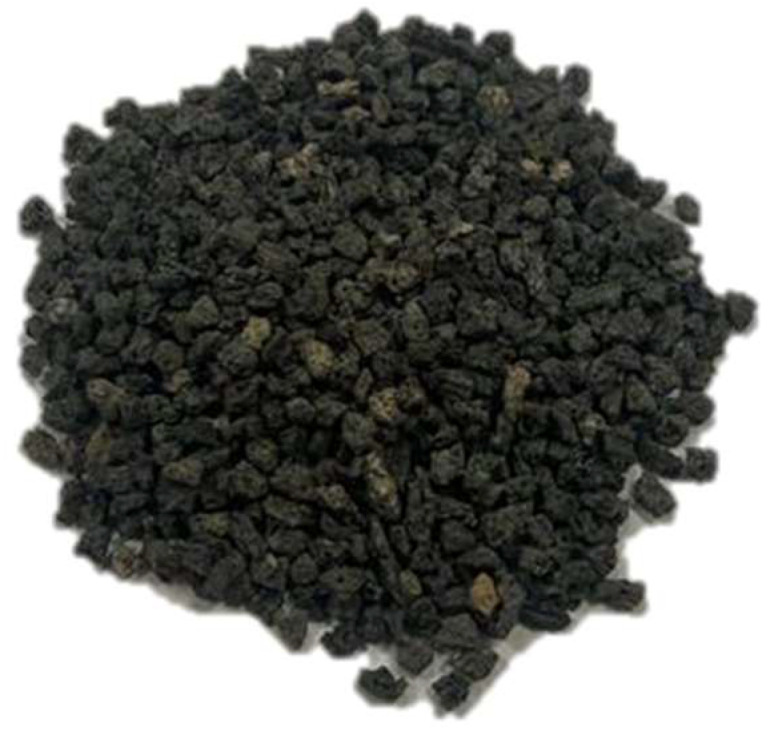
Pumice lightweight fine aggregate.

In making ultra-high-performance lightweight concrete mixtures, a binder was produced by combining Type I OPC and locally sourced silica fume (SF). The chemical constituents of both the OPC and SF can be found in Table 1. With a specific surface area of 17.5 m^2^/g, the silica fume used in this study exhibited considerable fineness. In the present study, single-sized, volcanic pumice was used as an LWA, devoid of contaminants that could potentially hinder the setting and hardening processes of the binder (see Figure 1). The aggregate was derived from a volcanic tuff of scoria found on the outskirts of Al-Medina, KSA. With a size of 4 mm and a density of 2.93 g/cm^3^, the fine aggregate demonstrated desirable properties for use in the project.

Selecting a single-sized aggregate aimed at guaranteeing concrete’s flowability and dispersion within the matrix is important. Achieving uniformity can be challenging when using graded aggregates in UHPLWC, mainly due to the limited amount of water employed in the mixture. To ensure optimal flowability in the UHPC mixtures while maintaining a relatively low dosage, a polycarboxylate ether-based additive was used as an admixture.

### 2.2. Development of Samples and Mix Design

Seven distinct concrete mixes with a uniform *w*/*b* of 0.21 were adopted with different percentages (0% to 30%) of lightweight aggregates fractionally substituting the fine aggregate developed. The complete details of all mixtures are presented in Table 2. The samples were characterized by a number and the letter “L”. The “L” indicates the lightweight aggregate, and the number shows the proportion of lightweight aggregates added in a sample as a substitute for the fine aggregate in the UHPC mix. Three samples were prepared, and their average value was taken as a final value.

A planetary mixing apparatus was employed for blending the various components. The dry ingredients, consisting of OPC, SF, quartz sand, and a lightweight pumice aggregate, were combined in the mixer and stirred slowly for three minutes. Following this, water and a superplasticizer were added to the blend, and the stirring process continued for an additional two minutes until the mixture became pliable.

Once the mixing process was complete, the mixes were discharged into various molds designed to create samples for compression and flexure strength evaluations. These molds were subsequently covered with a plastic sheet kept at an ambient temperature of 24 degrees Celsius for one day. Upon completion of the demolding process, the samples were submerged in water and cured for different numbers of days before their tests. Cylinder-shaped molds, with dimensions of 50 mm in diameter and 100 mm in length, were utilized for the compressive strength assessments, while prismatic molds, measuring 40 mm in width, 40 mm in depth, and 160 mm in length, were employed for the flexural strength evaluations. To investigate the effects of high temperatures on the properties of UHPC, the test specimens, which included cylinders and prisms, underwent 56 days of curing before being subjected to elevated temperatures of 100, 200, and 300 degrees Celsius. This temperature increase occurred within an electric furnace at 10 degrees Celsius per minute.

Each specimen was maintained at the specified temperature for two hours. Following this exposure, the samples were allowed to return to room temperature within the confines of the furnace before undergoing testing. This cooling process was crucial for accurately measuring the UHPC’s performance under heightened temperatures.

### 2.3. Characterization of Tests

The rheology was conducted on various fresh mixtures following the ASTM C143 [45] standard. A standard truncated cone featuring a 200 mm internal diameter at its base, a 100 mm diameter at its apex, and a 300 mm height was utilized during this process. The flow measurement was determined by averaging the diameters of the circularly spread concrete.

Compressive strength tests complied with ASTM C39 [46], while flexural strength tests were executed using the 3-point loading method per ASTM C78 [47]. Four duplicates were tried for each specimen type and property, with the results averaged. An electromechanical testing machine (Toni Technik) was employed for the strength tests, operating under load control at a 4 kN/s rate. This rate was precisely sustained until the load indicator displayed a decreasing trend and the samples exhibited distinct fracture patterns. The fracture patterns of each sample were carefully examined to discern the failure modes of the various samples. Additionally, the loss in mass of the UHPC’s samples, expressed as a (%), was calculated by evaluating the concrete’s mass before and after subjecting it to fire.

Mercury intrusion porosimetry (MIP) was employed for porosity and pore structure assessment. Before testing, it was ensured that samples were oven-dried at 60 degrees Celsius until a constant mass was attained to eliminate residual moisture content. MIP provides valuable information regarding pore size distribution, porosity, and pore connectivity, crucial to understanding the concrete’s durability and mechanical properties. Thermal analysis of the concrete was performed using thermogravimetric analysis (TGA) and differential scanning calorimetry (DSC). TGA helps determine mass loss due to thermal decomposition or phase transitions, while DSC measures the heat flow associated with these transitions. By combining both techniques, we can effectively assess the thermal stability, reaction kinetics, and potential thermal effects on ultra-high-performance lightweight concrete. X-ray diffraction (XRD) analysis was conducted to identify the crystalline phases within the concrete and evaluate the degree of hydration. XRD offers insights into the mineralogical composition and phase transformations, which are essential for understanding the material’s mechanical performance and long-term durability.

Lastly, for microstructural behavior, samples were assessed utilizing scanning electron microscopy (SEM). Remnant samples derived from the compressive strength tests were repurposed for SEM examination. Before testing, the samples were oven-dried at 60 degrees Celsius until a uniform mass was reached, halting further hydration.

## 3. Results and Discussion

### 3.1. Workability of UHPC

**Figure 2:**
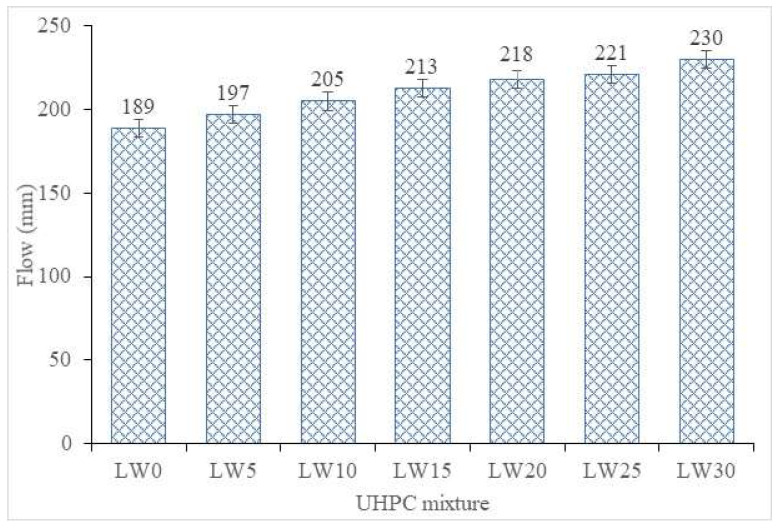
Workability of UHPC.

The test result of the flowability of all UHPC mixtures is presented in Figure 2. The addition of lightweight fine aggregates (pumice) in UHPC as a partial replacement for fine aggregates has been observed to impact the workability or flow of the resulting mixture significantly. As the percentage of lightweight aggregates was increased from 5% to 30%, the flowability of the UHPC increased correspondingly, with the mixture containing 30% lightweight aggregate displaying the highest flowability (230 mm) among all the mixtures tested.

The unique physical properties of the lightweight aggregate can explain this phenomenon. Lightweight aggregates have a lower specific gravity and higher porosity than traditional aggregates, reducing the mixture’s overall density [48]. The decreased density increases the volume of paste, which acts as a lubricant and helps facilitate ease of movement within the mixture. Additionally, the high porosity of the lightweight aggregate provides more surface area for the paste coating, leading to improved lubrication and ease of movement [49]. These characteristics ultimately enhance the flowability of the UHPC. The increase in the flowability of the UHPC can be further enhanced by increasing the percentage of the lightweight aggregate. This trend was observed in the mixture containing 30% lightweight aggregate, which had the highest flowability among all the mixtures tested. This increase in flowability may be attributed to the greater proportion of lightweight aggregate present in the mixture, which further reduces the density of the concrete and creates a greater volume of paste [50].

### 3.2. Density

**Figure 3:**
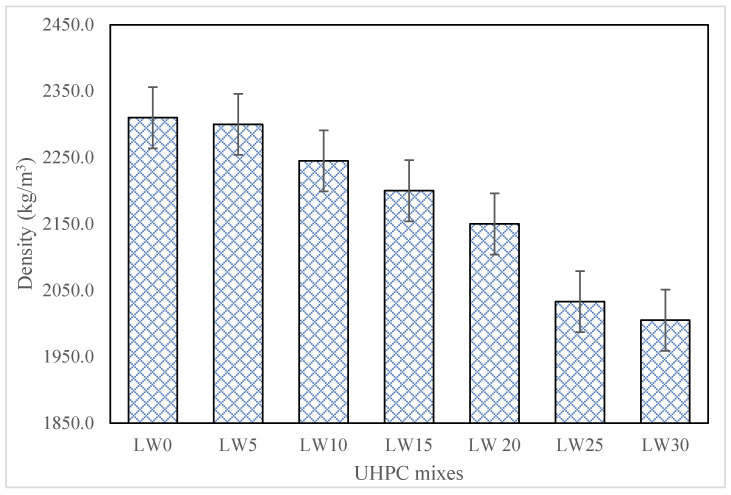
Hardened density of UHPC.

The use of lightweight fine aggregates (pumice) in UHPC as a partial substitute for traditional aggregates such as sand has been observed to result (See Figure 3) in a significant reduction in the overall density of the mixture. As the percentage of lightweight fine aggregates increased from 5% to 30%, the density of the UHPC decreased correspondingly, with the mixture containing 30% lightweight fine aggregates exhibiting the lowest density among all the mixtures tested. This reduction in density can be attributed to the unique physical properties of the lightweight aggregate. Compared to traditional aggregates, lightweight aggregates have a lower specific gravity and higher porosity, which results in a lower overall density of the mixture when used as a partial substitute [51]. Including lightweight fine aggregates reduces the total weight of the mixture while maintaining the same volume, ultimately resulting in a lower density of the UHPC. With the inclusion of 30% lightweight aggregate, the density of concrete reduced from 2310 kg/m^3^ to 2005 kg/m^3^. The reduction in density of the UHPC due to the inclusion of lightweight fine aggregates may have implications for the design of UHPC structures. For example, lower-density UHPC can reduce the overall weight of a structure, potentially leading to cost savings in construction and transportation [52,53].

Moreover, the high porosity of the lightweight fine aggregates allows for better mixing with the cement paste, creating a more homogenous mixture. The high surface area of the lightweight fine aggregates also results in a greater paste volume, further reducing the overall density of the UHPC [54]. As the percentage of lightweight fine aggregates was increased to 30%, the UHPC mixture had the lowest density among all the mixtures tested. This is because the higher proportion of lightweight fine aggregates in the mixture displaces heavier traditional aggregates, reducing the overall weight of the mixture while maintaining the same volume.

### 3.3. Mass Loss in UHPC

**Figure 4:**
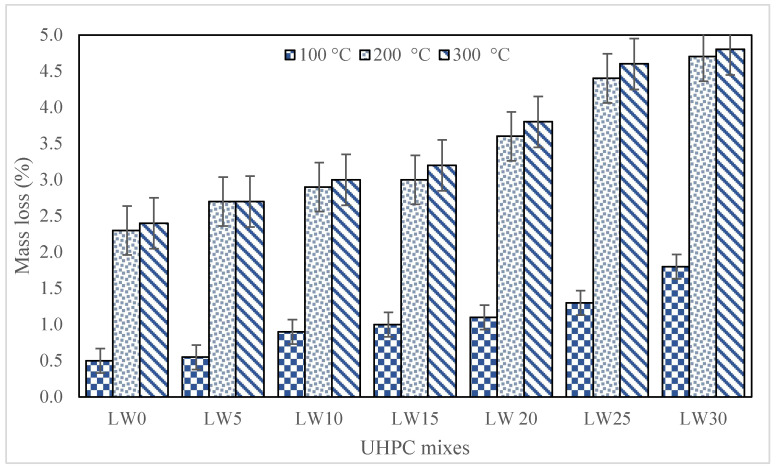
Mass loss in UHPC after exposure to elevated temperature.

The mass loss in ultra-high-performance lightweight aggregate concrete when subjected to 100 °C, 200 °C, and 300 °C is presented in Figure 4. Figure 4 shows that as the percentage of lightweight aggregates increased from 0% to 30%, the mass loss in the UHPC mixture also increased with increasing temperature. One possible explanation for why the mass loss in the UHPC was higher when the fine aggregates were replaced with a lightweight pumice aggregate from 0% to 30%, with the mixture containing 30% pumice having the highest mass loss, is that the lightweight aggregate may have lower thermal stability than the fine aggregates. At 300 °C, the LW30 had 4.8% mass loss, whereas LW0 had only 2.4%. When UHPC is exposed to high temperatures, the lightweight aggregate may break down faster than the fine aggregate, leading to more significant mass loss. This is because lightweight aggregates often have higher porosity, which allows them to absorb more moisture and other contaminants, making them more vulnerable to thermal degradation. UHPC is susceptible to damage when exposed to high temperatures [55]. When UHPC is exposed to temperatures above 100 degrees Celsius, it can experience thermal degradation, leading to mass loss, cracking, and other forms of damage. In addition to porosity, the chemical composition of the lightweight aggregate may also play a role in the higher mass loss observed in the UHPC mixtures containing the lightweight aggregate [56]. Pumice, for example, is a volcanic rock that contains significant amounts of water and other volatile compounds. When exposed to high temperatures, these compounds may be released from the lightweight aggregate, contributing to the mass loss of the UHPC [57]. Another factor that may contribute to the higher mass loss in the UHPC containing the lightweight aggregate is the difference in thermal expansion between the lightweight and fine aggregates. When UHPC is exposed to high temperatures, the different thermal expansion rates between the two aggregates can lead to internal stresses, which can cause cracking and other damage [58].

### 3.4. Compressive Strength

**Figure 5:**
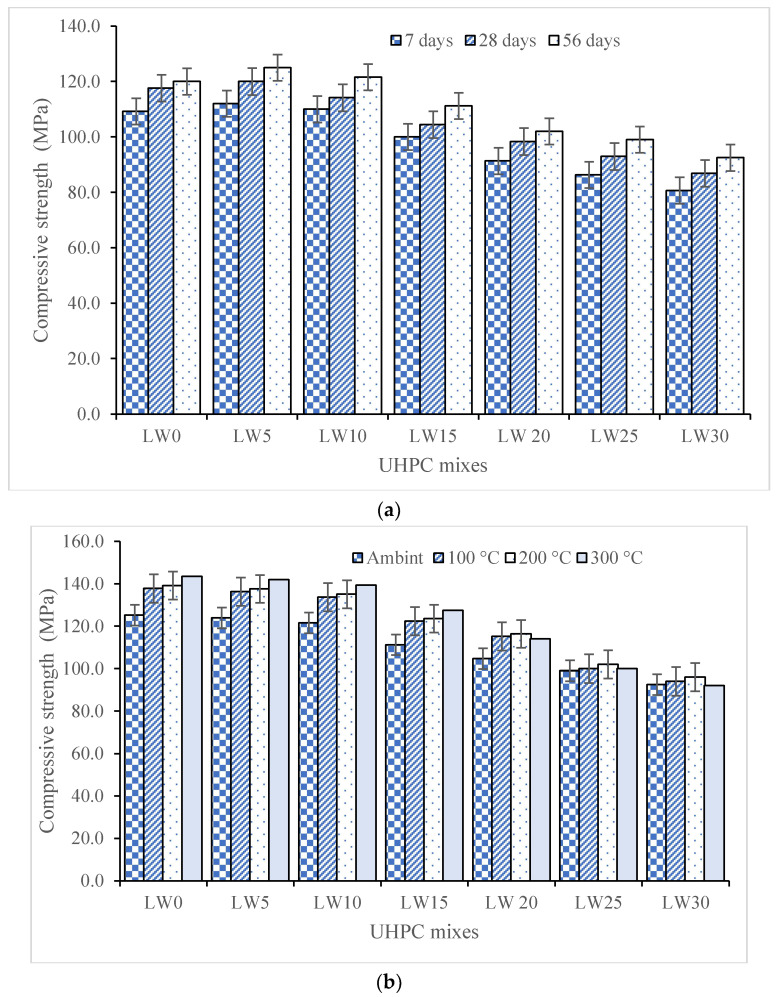
Compressive strength of UHPC: (**a**) at 7, 28, and 56 days of curing; (**b**) at ambient and after elevated temperature.

The compressive strength of the UHPC with different percentages of LWAs at the curing of 7, 28, and 56 days is presented in Figure 5a. The test results show that as the percentage of lightweight aggregates increases from 0% to 30%, the compressive strength reduces at every corresponding curing duration. For 5% and 10% LWA at 56 days, the compressive strength was observed to increase by 4% and 1.31% compared to the sample with 0% LWA, but after that, the compressive strength was continuously reduced. The reduction in the compressive strength of the UHPC as the fine aggregates were replaced with lightweight aggregates can be attributed to several scientific reasons.

Pumice aggregates have a lower density compared to the fine aggregates typically used in UHPC, which can lead to a lower packing density of the concrete mixture. This reduced packing density can result in a higher porosity of the concrete, which can negatively impact the strength and durability of the concrete. In addition, pumice aggregates have a lower modulus of elasticity than fine aggregates, which can result in higher deformation and cracking of the concrete under load. This can also contribute to the reduction in the compressive strength of UHPC with higher percentages of pumice aggregates. In the present study, the compressive strength at 56 days reduced from 120 MPa to 92.5 MPa when up to 30% LWA was added. Furthermore, pumice aggregates have a lower surface area and surface roughness than fine aggregates, leading to a weaker bond between the aggregate and the cement paste [59]. This weaker bond can result in a lower interfacial transition zone (ITZ) strength, which can further reduce the compressive strength of the UHPC.

Several research studies have studied the reduction in compressive strength of UHPC due to the replacement of fine aggregates with lightweight pumice aggregates. In a study conducted by Gündüz [60], it was found that the compressive strength of concrete decreased as the percentage of pumice aggregates increased. The study concluded that the reduction in compressive strength was mainly due to the lower density of pumice aggregates, which resulted in a higher porosity of the concrete and a weaker ITZ [61]. Another study conducted by Meng et al. (2023) [62] investigated the effect of pumice aggregates on the mechanical properties of UHPC. The study found that the compressive strength of UHPC decreased as the percentage of pumice aggregates increased, with the mixture containing 30% pumice aggregate exhibiting the lowest compressive strength on each curing day. The study also found that adding pumice aggregates led to increased water absorption and total porosity of the UHPC, which further contributed to the reduction in compressive strength.

Figure 5b displays the compressive strength of the UHPC containing varying percentages of the LWA when subjected to elevated temperatures (100 °C, 200 °C, and 300 °C). The compressive strength of the UHPC with the lightweight aggregate was reduced at all curing days (7, 28, and 56) when exposed to elevated temperatures of 100, 200, and 300 degrees Celsius. However, simultaneously, the samples subjected to elevated temperatures had higher compressive strength than those with ambient conditions at every replacement level of fine aggregates. At LW30, the sample exposed to 200 °C had 96 MPa strength, while the sample with the ambient condition had 92.5 MPa compressive strength. Different mechanisms can explain this contradictory behavior. One possible reason for the reduction in compressive strength of the UHPC with the lightweight aggregate at elevated temperatures is the thermal degradation of the aggregate [63]. As pumice is a volcanic rock, it may undergo phase transformation or breakdown when exposed to high temperatures, resulting in a weakened aggregate [64]. This, in turn, can reduce the strength of the UHPC. On the other hand, the increased compressive strength observed in the samples subjected to elevated temperatures can be attributed to the accelerated rate of chemical reactions in concrete at high temperatures [65]. As mentioned earlier, the higher activity of water molecules at elevated temperatures results in a faster reaction between the cementitious materials and the fine aggregates. This leads to increased cement hydration and, subsequently, higher compressive strength [66].

### 3.5. Flexure Strength

**Figure 6:**
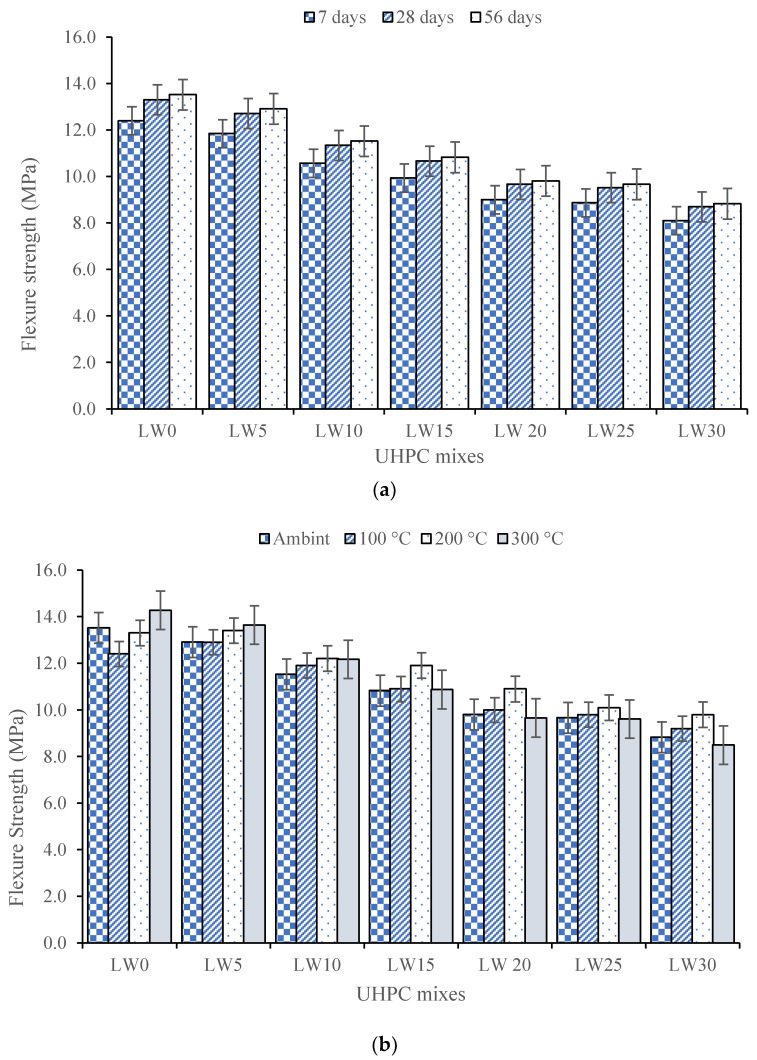
Flexural strength of UHPC: (**a**) at 7, 28, and 56 days of curing; (**b**) at ambient and after elevated temperature.

Figure 6a illustrates the flexural strength of UHPC samples with varying percentages of lightweight aggregates at curing durations of 7, 28, and 56 days. The test outcomes indicate that as the LWA percentage increases from 0% to 30%, there is a corresponding reduction in flexural strength at each curing duration.

At 56 days of curing, the flexure strength was reduced by 34.81% when the LWA was increased from 0% to 30%. The reduction in flexure strength of the UHPC containing 0% to 30% of lightweight pumice aggregates at 7, 28, and 56 days of curing can be attributed to several factors. Firstly, lightweight aggregates have lower mechanical properties compared to conventional aggregates. The modulus of elasticity and compressive strength of lightweight pumice aggregates are lower than those of traditional aggregates, resulting in a reduction in the composite strength of the UHPC. The reduced strength of the lightweight aggregates can also lead to a reduction in the load-carrying capacity of the composite [67]. Secondly, the lower bonding strength between the cement matrix and the lightweight aggregates can decrease the interfacial bond strength between the aggregates and the cement paste [68]. The lightweight aggregates have a lower surface area and higher porosity compared to conventional aggregates, which can lead to a weaker bonding between the cement matrix and the lightweight aggregates. This can result in a reduction in the flexural strength of the UHPC. Thirdly, incorporating lightweight aggregates in UHPC can increase the composite’s porosity, reducing its density and strength. The higher porosity of the lightweight aggregates can result in a higher volume of air voids in the composite, reducing the load-carrying capacity of the UHPC. Finally, the reduction in flexure strength observed in the UHPC sample with 30% lightweight aggregates can be attributed to the combination of the above factors. The higher percentage of lightweight aggregates in the UHPC resulted in a higher volume of air voids, weaker bonding between the cement matrix and the lightweight aggregates, and a lower load-carrying capacity, significantly reducing the flexure strength [69].

The flexural strength of the ultra-high-performance concrete with different percentages of the lightweight aggregate when exposed to elevated temperatures of 100 °C, 200 °C, and 300 °C is presented in Figure 6b. The reduction in the flexural strength of the ultra-high-performance concrete containing 0% to 30% of a lightweight (pumice) aggregate when subjected to elevated temperatures of 100, 200, and 300 degrees Celsius can be attributed to the thermal degradation of the cement paste and the lightweight aggregate. The hydration products in the cement paste, such as C-S-H gel and calcium hydroxide, undergo thermal decomposition at elevated temperatures, reducing the strength of the UHPC [70]. At LW30, the sample exposed to 200 °C had 9.8 MPa flexure strength, while the sample with the ambient condition had 8.8 MPa flexure strength (10.2% higher flexure strength at 200 °C). Similarly, the lightweight pumice aggregate undergoes thermal expansion due to the presence of water and other volatile compounds, which can result in microcracks and a reduction in the load-carrying capacity of the composite [71]. However, the samples subjected to elevated temperatures had higher flexural strength than those with ambient conditions at every replacement level of fine aggregates. This can be attributed to the thermal curing of the UHPC at elevated temperatures, which can result in accelerated strength gain due to the increased rate of hydration of the cementitious materials [72]. The high temperature can also lead to additional C-S-H gel, enhancing the bond between the cement matrix and the lightweight aggregate and higher flexural strength [73].

Furthermore, the higher flexural strength observed in the samples subjected to elevated temperatures can also be attributed to the reduction in the porosity of the UHPC at high temperatures. The heating can cause water evaporation from the UHPC, resulting in a denser and stronger composite. The reduction in porosity can also lead to a lower volume of air voids, reducing the susceptibility of the UHPC to thermal cracking [74].

### 3.6. Porosity

**Figure 7:**
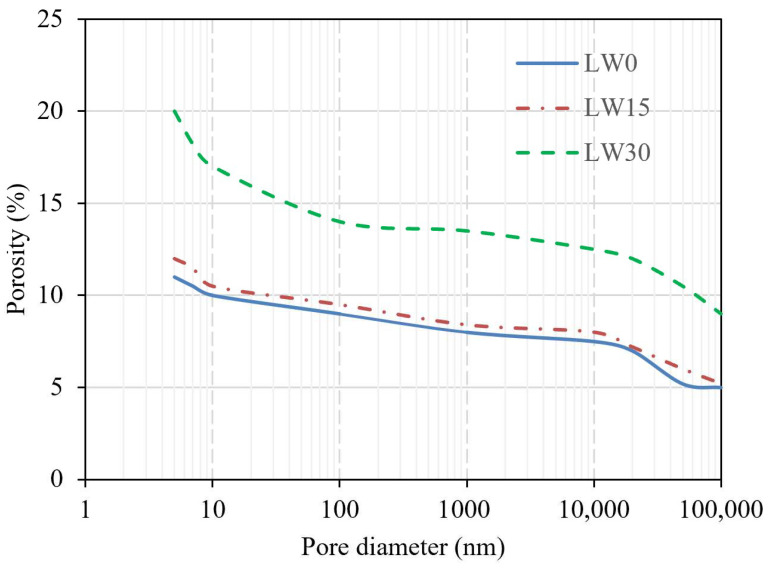
Effect of LWA on the porosity of UHPC after hydrating at 56 days.

The porosity of UHPC with different percentages of lightweight aggregates at 56 days is shown in Figure 7. In the UHPC samples containing varying concentrations of lightweight pumice aggregates, it was observed that at the hydration of 56 days, the porosity of the composite was higher for the sample with 30% lightweight aggregates compared to the samples with 0% and 15% lightweight aggregates. This increase in porosity can be primarily attributed to the intrinsic porous nature of the pumice, a volcanic rock with a high volume of voids resulting from the rapid cooling and solidification of lava [75]. Using pumice as a partial replacement for traditional fine aggregates in a UHPC mix increases the overall porosity of the composite due to incorporating these additional voids originating from the lightweight aggregates [76]. However, in the case of the sample with 15% lightweight aggregates, the lowest porosity compared to 30% lightweight aggregates was observed and was almost similar to samples with no lightweight aggregates. This captivating phenomenon can be explained by considering several factors that may have contributed to this result. One potential explanation is the synergistic effect of the pumice particles and the cementitious matrix, which may have enhanced the composite’s packing density and microstructural refinement [77]. The partial replacement of traditional aggregates with a smaller proportion of pumice (15%) might have resulted in an optimal balance between the packing density and void content within the composite, producing a denser microstructure [78]. Another factor to consider is the influence of the pumice aggregates on the water absorption and release during the mixing and hydration processes. The porous nature of pumice allows it to absorb a significant amount of water, which may be gradually released into the cementitious matrix during hydration [79]. The water released by the pumice particles might have contributed to a more efficient hydration process, ultimately resulting in a denser and less porous microstructure in the 15% LWA sample.

Additionally, the 15% lightweight aggregate content may have led to a more favorable pore size distribution within the UHPC matrix. A more heterogeneous pore size distribution, with smaller and larger pores, can result in a more tortuous pore structure that impedes the ingress of aggressive agents and enhances the material’s durability.

### 3.7. Pore Structure

**Figure 8:**
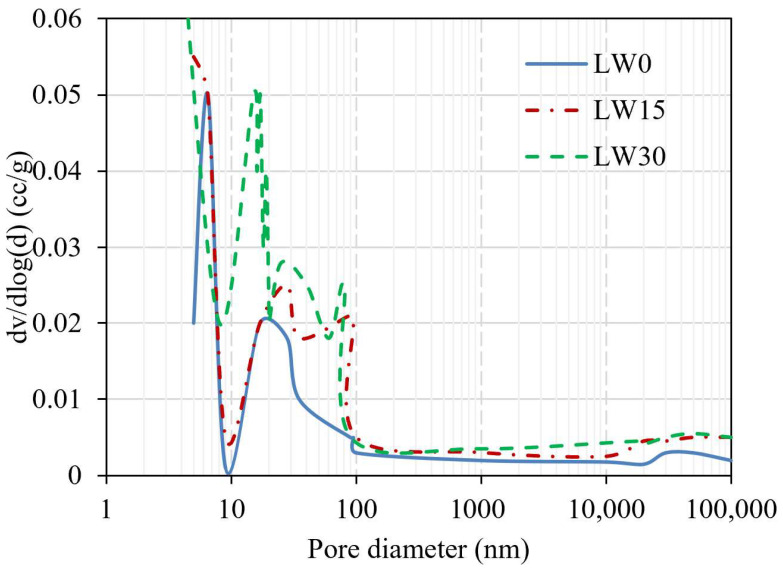
Effect of LW content on the pore size distribution.

In Figure 8, the graph’s *x*-axis represents the pore diameter, which ranges from 1 to 100,000, and the *y*-axis represents dV/dlog(d) (cc/g), which measures the material’s pore volume per unit mass. The pore structure of UHPC is an essential factor in determining its mechanical properties, particularly its strength and durability. The graph shows three lines, each representing a different percentage of lightweight aggregates in the UHPC mix. The first line defines the UHPC mix without any lightweight aggregates (0%), the second line represents the mix with 15% lightweight aggregates, and the third line represents the mix with 30% lightweight aggregates. The lines intersect at various points on the graph, indicating changes in the pore structure as the percentage of lightweight aggregates in the mix changes. The points of intersection can be used to identify the threshold at which the addition of lightweight aggregates begins to impact the pore structure of the UHPC significantly. By analyzing the trends and points of intersection of the lines, important insights can be drawn about the effects of lightweight aggregates on the pore structure of the UHPC. For example, the graph reveals that the addition of lightweight aggregates increases the overall porosity of the UHPC but that there is a limit beyond which further additions have diminishing returns [80]. Overall, Figure 8 provides a visual representation of the impact of different amounts of lightweight aggregates on the pore structure of the UHPC, which is a critical factor in the material’s strength and durability. The results of this study can have important implications for developing new, high-performance concrete materials with improved properties, particularly in applications where weight reduction is a crucial consideration [81].

### 3.8. Shrinkage

**Figure 9:**
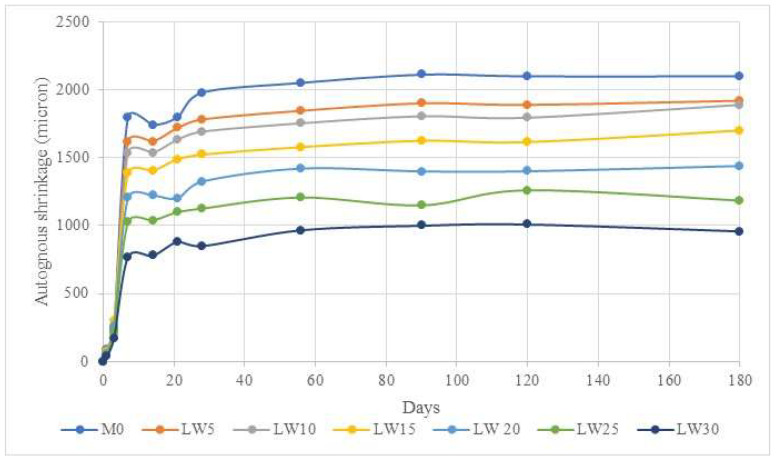
Effect of LWA on shrinkage of UHPC.

The effect of replacing fine aggregates with lightweight pumice aggregates on the shrinkage of the UHPC is displayed in Figure 9. The test result showed that increasing the percentage of LWAs reduced the shrinkage of the UHPC. The reduction in autogenous shrinkage of the ultra-high-performance concrete as the percentage of lightweight aggregates increased can be attributed to several factors. Autogenous shrinkage is a phenomenon that occurs in concrete due to autogenous drying, which is caused by the ongoing hydration reactions in the cement paste. This phenomenon can lead to the development of tensile stresses in the concrete, which can cause cracking and other forms of damage [82]. Adding LWAs as a partial substitute for fine aggregates in UHPC can help mitigate this problem by reducing the amount of cement paste in the mix and the overall volume changes during hydration. This reduction in the cement paste content results in lower internal relative humidity and a decrease in the amount of water available for autogenous drying, which in turn leads to a reduction in autogenous shrinkage [10]. The presence of lightweight aggregates in the UHPC mix also provides a better internal drainage system, which allows the excess water to escape more quickly, reducing the overall shrinkage of the concrete. This improved drainage is due to the larger pores in the LWA, which can help to transport the excess water more efficiently than the smaller pores in the cement paste. This enhanced drainage can further reduce the amount of internal moisture available for self-desiccation and minimize the risk of cracking [83]. In addition, using a LWA in UHPC can also improve the material’s durability by reducing the concrete’s permeability. This is because the LWA acts as a barrier to the penetration of water and other harmful substances, preventing them from reaching the internal surfaces of the concrete and causing damage [84]. The study results showed that the sample with 0% LWA had the highest shrinkage (2100 microns) at all corresponding curing days, while the sample with 30% LWA had the lowermost (958 microns) shrinkage across all curing days. This highlights the beneficial effect of LWAs on the autogenous shrinkage of UHPC and provides valuable insights into using LWAs as a partial substitute for fine aggregates in UHPC to improve its properties.

### 3.9. Thermal Analysis

**Figure 10:**
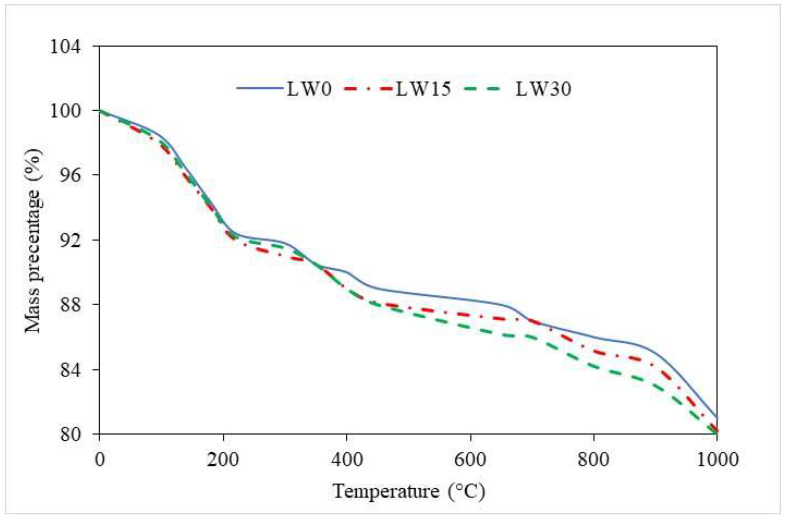
Thermal analysis of UHPC.

The thermal analysis of the UHPC with lightweight pumice aggregates under different temperatures is displayed in Figure 10. The thermal behavior of the materials can explain the reduction in the percentage of the mass of the UHPC with an increase in the percentage of LWAs as a partial substitute for fine aggregates. When the temperature increases, the lightweight aggregates experience a higher thermal expansion rate than the other components of the UHPC mixture. This leads to a decrease in the density of the UHPC, reducing the mass percentage of the material.

Furthermore, the decrease in the mass percentage of the UHPC mixture with an increase in LWA percentage is observed across all curing days, indicating that this effect is not limited to a specific point in the curing process. This is likely because the thermal properties of the LWA are not affected significantly by the curing process [85]. Therefore, the reduction in mass percentage observed at higher temperatures is consistent throughout the curing period. The sample with 0% LWA had the highest mass percentage at all corresponding temperatures, indicating that the absence of lightweight aggregates leads to a denser UHPC mixture. On the other hand, the sample with 30% LWA had the lowest mass percentage compared to LW0 and LW15 across all curing days, indicating that a high percentage of LWAs leads to a less dense UHPC mixture [86].

### 3.10. X-ray Diffraction Analysis

The XRD spectra show the diffraction pattern of the crystalline phases present in the material. The peak intensities in the spectra indicate the relative abundance of the different phases in each sample.

**Figure 11:**
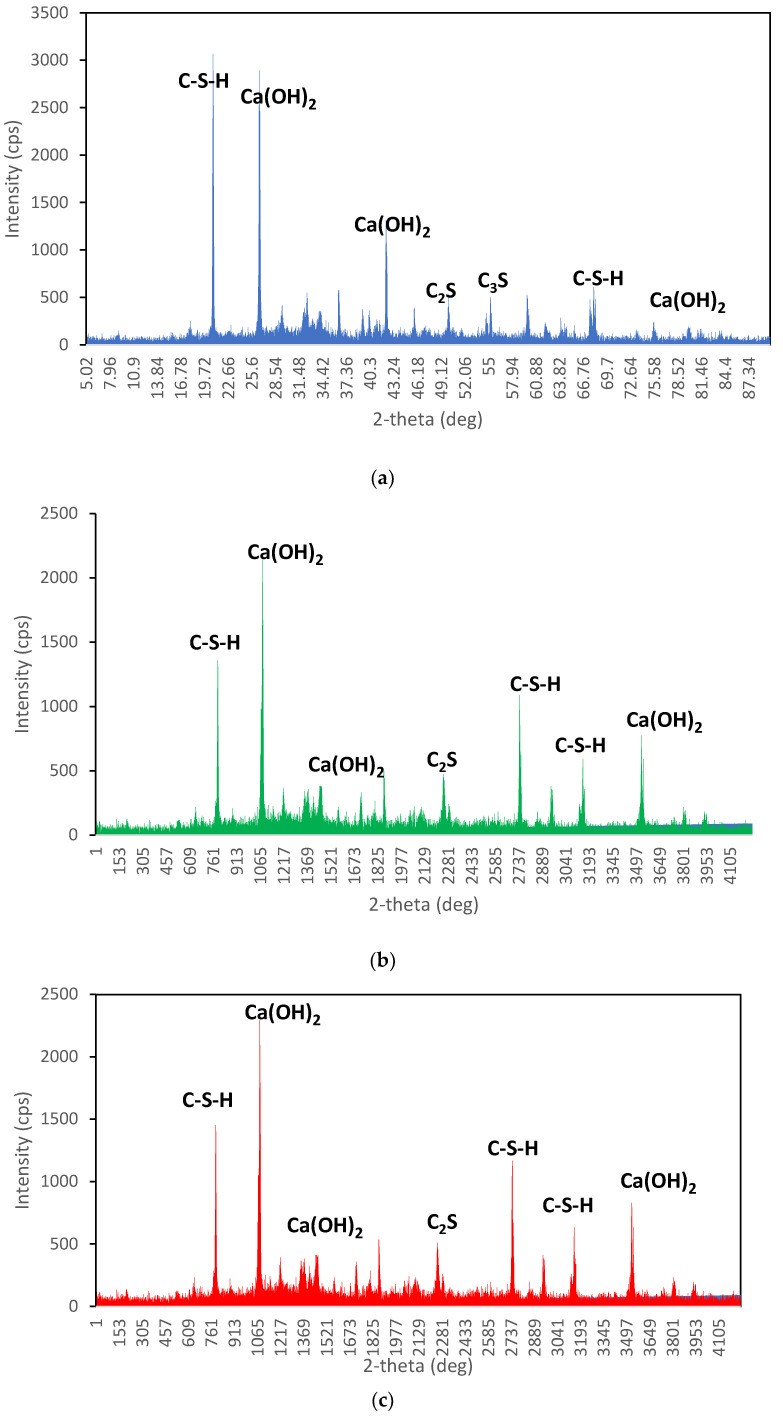
XRD spectra of UHPC: (**a**) LW0, (**b**) LW15, (**c**) LW30.

The first XRD spectrum (see Figure 11a), which corresponds to the UHPC sample with 0% replacement of fine aggregates with pumice, shows the highest peaks of portlandite (Ca(OH)_2_), calcium-silicate-hydrate (C-S-H), and ettringite intensities. This suggests that the UHPC sample with no replacement of fine aggregates has higher crystallinity than the other samples. Sharp and intense peaks in the spectrum may indicate the presence of highly crystalline phases in the material [87], such as portlandite, a typical phase in cement-based materials. The second XRD spectrum (see Figure 11b), which corresponds to the UHPC sample with 15% replacement of fine aggregates with pumice, shows lower peak portlandite, C-S-H, and ettringite intensities than the first spectrum. This suggests that replacing fine aggregates with pumice has decreased the crystallinity of the UHPC. Some of the same peaks as the first spectrum indicate that the UHPC sample with 15% replacement still contains some of the same crystalline phases as the UHPC sample with 0% replacement. The third XRD spectrum (see Figure 11c), which corresponds to the UHPC sample with 30% replacement of fine aggregates with pumice, shows an even lower peak of portlandite, calcium-silicate-hydrate, and ettringite intensities than the second spectrum. This suggests that replacing fine aggregates with pumice has further decreased the crystallinity of the UHPC [88]. Fewer peaks in the spectrum compared to the first two spectra may indicate that the UHPC sample with 30% replacement contains fewer highly crystalline phases.

### 3.11. Scanning Electron Microscopic Analysis

**Figure 12:**
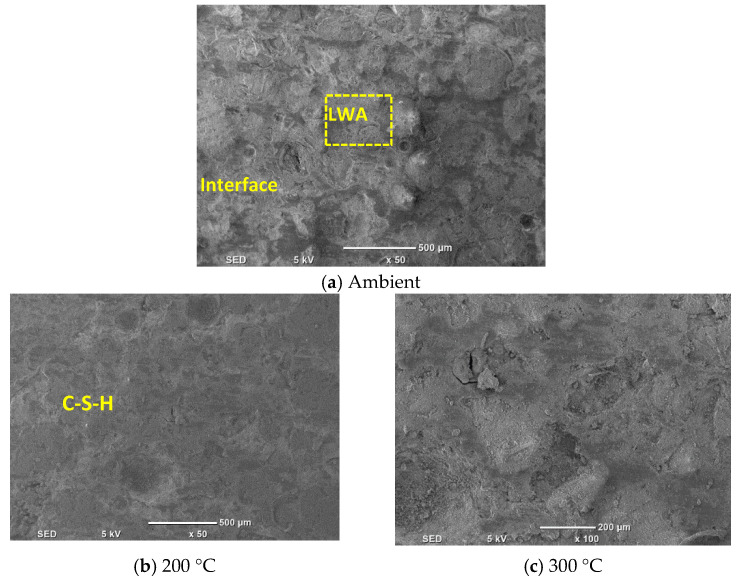
SEM analysis of UHPC (**a**–**c**).

**Figure 13:**
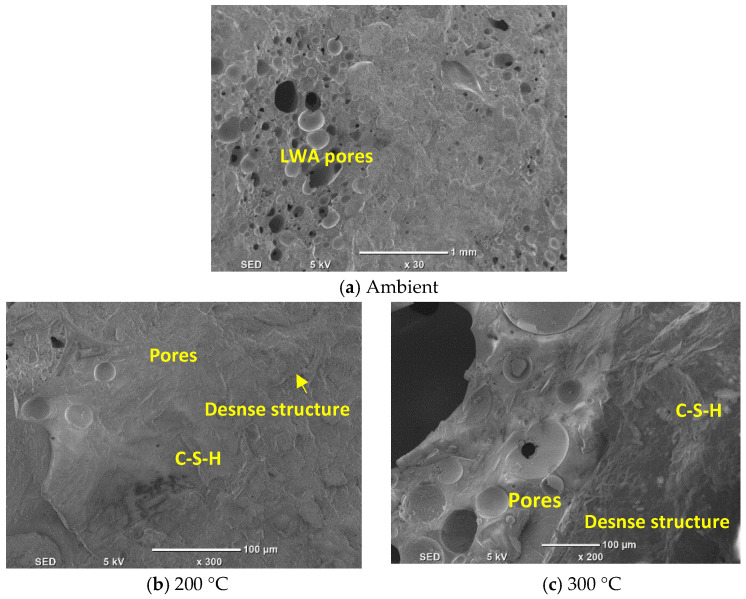
SEM analysis of UHPC with LWA (**a**–**c**).

SEM micrographs of UHPC specimens containing 0% and 30% LWA, both heated and unheated, have been analyzed and are presented in Figure 12 and Figure 13. SEM is a commonly used technique for examining the microstructure of concrete, particularly UHPC, due to its high resolution and ability to capture fine details.

Upon inspection of Figure 12a, it can be seen that the unheated UHPC specimen containing 0% LWA has a complex internal microstructure. The SEM image reveals the presence of various constituents, such as hydration products, un-hydrated cementitious material particles, aggregates, pores, and air voids. The main hydration products identified are calcium silicate hydrate gels and calcium hydroxide crystals [89]. The compact interface between the aggregate and matrix is particularly noteworthy, suggesting strong bonding between the two phases. The SEM image also shows the presence of relatively larger pores, which could be attributed to incomplete filling of the voids during casting or bleeding of the mixture. When the UHPC specimen is heated, the microstructure changes significantly. The SEM image in Figure 12b,c shows a more homogeneous microstructure with fewer visible pores and a denser matrix. The reduction in porosity and increased density can be attributed to the heat treatment, which results in further hydration of the cementitious materials and improved bonding between the matrix and aggregates [90]. The densification of the microstructure leads to higher strength and durability of the UHPC. The SEM images of the UHPC specimens containing 30% LWA (Figure 13) show a similar trend to those with 0% LWA. The unheated specimen (Figure 13a) displays a more heterogeneous microstructure compared to the heated specimen (Figure 13a,b), with a range of particle sizes and shapes visible in the matrix. However, in contrast to the specimen with 0% LWA, the presence of the LWA particles is visible in the SEM images. The LWA particles are observed to have a distinct morphology and are generally well distributed within the matrix.

The SEM images in Figure 13 provide insights into the microstructure of UHPC specimens containing varying percentages of the LWA. The specimens with 30% LWA have a denser microstructure than those with 0% LWA, with fewer visible voids and cracks. The microstructure of the paste and the interface between the aggregate and paste appear to be more uniformly distributed in the specimens containing the LWA. This observation can be attributed to the continuous hydration process triggered by the supply of water stored in the LWA. The LWA particles act as internal curing agents by releasing water gradually, which ensures the continuous hydration of cementitious materials, resulting in a denser microstructure and improved performance of the UHPC [91]. However, the specimens with 15% LWA exhibit weaker performance than those with 0% LWA. This is likely due to the high porosity of the LWA particles, which can reduce the overall strength of the UHPC. The porosity of the LWA particles is an intrinsic weakness that can significantly affect the strength-reduction effect of concrete, particularly when present in a significant quantity. To overcome this issue, heating the UHPC specimens effectively improves their microstructure and compressive strength, as shown in Figure 13b,c. The elevated temperature during heating triggers further hydration of cementitious materials and leads to better bonding between the matrix and aggregates. This densification of the microstructure improves the strength and durability of the UHPC containing the LWA.

## 4. Conclusions

The present research assessed the effect of lightweight aggregates as a partial replacement of fine aggregates on ultra-high-performance concrete. Different strength, durability, and microstructural properties of the UHPC were evaluated. The following conclusions are obtained from the present research:

- Raising lightweight aggregates from 5% to 30% improves UHPC flowability, with the 30% mixtures showcasing exceptional flowability. This remarkable increase is due to the unique physical properties of lightweight aggregates.
- As lightweight fine aggregates increased from 5% to 30%, the UHPC density decreased, with 30% mixtures showing the lowest density. Lightweight aggregates’ lower specific gravity and higher porosity reduced overall density. Including 30% lightweight aggregates lowered the concrete density from 2310 kg/m^3^ to 2005 kg/m^3^.
- The test results indicate that increasing lightweight aggregates from 0% to 30% reduces compressive strength at all curing durations. At 56 days, the 5% and 10% LWA samples increased strength by 4% and 1.31% compared to the 0% LWA, but subsequently declined.
- The UHPC with the lightweight pumice aggregate had reduced compressive strength at all curing days and elevated temperatures. Yet, samples exposed to higher temperatures showed greater strength than ambient conditions at every replacement level. At LW30, 200 °C yielded 96 MPa, while an ambient temperature achieved 92.5 MPa.
- The flexure tests show that increasing the LWA percentage from 0% to 30% reduces flexural strength at each curing duration. At 56 days, flexure strength dropped by 34.81% from the 0% to 30% LWA. The LW30 samples exposed to 200 °C had 9.8 MPa flexural strength, 10.2% higher than the ambient-condition samples at 8.8 MPa.
- The observation at 56 days of hydration revealed that the composite porosity was higher for the sample containing 30% lightweight aggregates than those with 0% and 15% lightweight aggregates.
- The UHPC mass reduction with increased lightweight aggregate percentage is due to thermal behavior. Lightweight aggregates exhibit higher thermal expansion, decreasing the UHPC density and mass.
- The XRD spectra reveal that the UHPC crystallinity decreases with pumice replacing fine aggregates. The 0% pumice sample has the highest crystallinity, shown by peak portlandite, calcium-silicate-hydrate, and ettringite intensities. With 15% and 30% pumice replacements, the crystallinity and peak intensities decrease, indicating fewer highly crystalline phases.
- The SEM analysis of the UHPC specimens with varying percentages of the LWA reveals changes in their microstructure. The samples with 30% LWA have a denser microstructure due to the continuous hydration triggered by water stored in the LWA, resulting in improved paste performance near the aggregates.
- Heating the UHPC specimens improves their microstructure, triggering further hydration and better bonding between matrix and aggregates and improving strength and durability.

## Figures and Tables

**Table 1:** Chemical properties of OPC and SF.

	Content, %	
Oxide, %	OPC	Silica Fume
Silicon Dioxide (SiO_2_)	23	98.9
Calcium Oxide (CaO)	63.5	0.1
Aluminum Oxide (Al_2_O_3_)	4.5	0.1
Ferric Oxide (Fe_2_O_3_)	3.6	0.1
Magnesium Oxide (MgO)	2.3	0.1
Sulfur Trioxide (SO_3_)	2.1	0.1
Sodium Oxide (Na_2_O)	0.3	0.1
Potassium Oxide (K_2_O)	0.2	0.1
Calcium Sulfate (CaSO_4_)	0.4	N/A
Loss on Ignition	0.1	0.4

**Table 2:** Mix design of complete mixtures (kg/m^3^).

Mix ID	Cement	Silica Fume	Water	Sand	LWA	HRWR	Steel Fiber
LW0	900	221	192	990	0	30	78
LW5	900	221	192	950	41	30	78
LW10	900	221	192	909	81	30	78
LW15	900	221	192	869	122	30	78
LW20	900	221	192	828	162	30	78
LW25	900	221	192	788	203	30	78
LW30	900	221	192	828	162	30	78

## Data Availability

Not applicable.
